# Neurophysiological measures of auditory sensory processing are associated with adaptive behavior in children with Autism Spectrum Disorder

**DOI:** 10.1186/s11689-023-09480-2

**Published:** 2023-04-01

**Authors:** Mairin Cotter, Seydanur Reisli, Ana Alves Francisco, Kathryn-Mary Wakim, Leona Oakes, Michael J. Crosse, John J. Foxe, Sophie Molholm

**Affiliations:** 1 The Cognitive Neurophysiology Laboratory, Department of Pediatrics, Albert Einstein College of Medicine, Bronx, NY 10461 USA; 2 Department of Psychology, Fordham University, Bronx, NY 10458 USA; 3 Department of Neuroscience, Albert Einstein College of Medicine, Bronx, NY 10461 USA; 4 The Frederick J. and Marion A. Schindler Cognitive Neurophysiology Laboratory, The Ernest J. Del Monte Institute for Neuroscience, Department of Neuroscience, University of Rochester School of Medicine and Dentistry, Rochester, NY 14642 USA; 5 Segotia, Galway, Ireland; 6 Trinity Centre for Biomedical Engineering, Trinity College Dublin, Dublin, Ireland; 7 Department of Psychiatry and Behavioral Sciences, Albert Einstein College of Medicine, Bronx, NY 10461 USA

**Keywords:** Autism Spectrum Disorder, Electroencephalography, Adaptive behavior, Event related potentials, Lateralization, Auditory processing

## Abstract

**Background:**

Atypical auditory cortical processing is consistently found in scalp electrophysiological and magnetoencephalographic studies of Autism Spectrum Disorder (ASD), and may provide a marker of neuropathological brain development. However, the relationship between atypical cortical processing of auditory information and adaptive behavior in ASD is not yet well understood.

**Methods:**

We sought to test the hypothesis that early (100-175 ms) auditory processing in ASD is related to everyday adaptive behavior through the examination of auditory event-related potentials (AEPs) in response to simple tones and Vineland Adaptive Behavior Scales in a large cohort of children with ASD (*N* = 84), aged 6–17, and in age- and IQ- matched neurotypically (NT) developing controls (*N* = 132).

**Results:**

Statistical analyses revealed significant group differences in early AEPs over temporal scalp regions (150-175 ms), and the expected rightward lateralization of the AEP (100-125 ms and 150-175 ms) to tonal stimuli in both groups. Lateralization of the AEP (150-175 ms) was significantly associated with adaptive functioning in the socialization domain.

**Conclusions:**

These results lend support to the hypothesis that atypical processing of sensory information is related to everyday adaptive behavior in autism.

## Background

Cortical sensory processing differences in Autism Spectrum Disorder (ASD) may be indicative of aberrant neurodevelopment, and are likely to have cascading effects on higher order cognitive processes [35] that in turn impact clinical phenotype. Studies using electrophysiological (EEG) recordings to examine the brain response to auditory stimulation in ASD consistently reveal smaller and/or slightly delayed auditory evoked potentials (AEP for EEG recordings; auditory evoked magnetic fields [AEMF] for magnetoencephalographic [MEG] recordings) 100–200 ms post stimulus onset over frontal and lateral temporal scalp regions in comparison to age-matched neurotypical (NT) controls [6, 7, 27, 48, 70, 75]. As such AEPs present a strong candidate for a neural marker of cognitive, clinical, and behavioral sequelae of ASD.

Prior work has been directed at exploring relationships between atypical AEPs and the autism phenotype [5, 19], yet very few studies have focused on the relationship between cortical auditory sensory processing and how well a child with a diagnosis of ASD is able to navigate age-appropriate everyday situations (“adaptive behavior”). The Vineland Adaptive Behavior Scales (Vineland) provide an age appropriate measurement of adaptive behavior in the areas of socialization, communication, daily living, motor skills, and maladaptive behavior [69], and can be used to represent the impact of a neurodevelopmental condition on daily living [8]. Focusing on the communication domain of adaptive behavior, Roberts and colleagues [41, 42, 60] found that the latency of the early auditory MEG response to tonal stimuli was correlated with Vineland adaptive communication scores in a sample of ASD and NT children. Here, we sought to further explore the relationship between auditory processing in ASD and adaptive behavior, by evaluating the relationship between the Vineland domains of socialization and daily living skills in addition to the domain of communication in a large sample of children and adolescents with ASD, using high-density EEG to index auditory sensory processing.

Brain activity in response to tonal and musical stimuli is typically stronger in the right compared to the left cortical hemisphere [37, 41, 42, 47, 50, 60], whereas this pattern is reversed in response to speech and language stimuli [26, 30, 44]. Lateralization of cortical function is observed in many functional domains in humans [14, 24, 64], and is often reduced or altered in neurodevelopmental and neuropsychiatric conditions [3, 4, 11, 23, 55, 59, 74]. Furthermore, differences in cortical network asymmetries are seen in infants at risk for ASD [62] as well as in sensory processing regions in infants that later go on to receive a diagnosis of ASD [35] and there is extensive evidence for reduced lateralization of language/speech processing in ASD [18, 38] and in at risk 6–12 month old infants [67]. Studies similarly suggest diminished rightward lateralization for non-speech stimuli in ASD, although to date this has not been extensively reported on [13, 21, 28, 41, 42, 47, 60, 65, 75]. The relationship between auditory lateralization of brain responses to tones and adaptive behavior, however, has not been previously considered.

Here we examined the N1 response of the AEP to simple tones in a cohort of 84 ASD and 132 control participants, ranging in age from 6 to 17, and considered how these responses were related to adaptive behavior. The auditory N1 can be parsed into subcomponents with positive and negative deflections peaking between ~ 70 and 175 ms and with foci over temporal and frontocentral scalp regions. The responses over temporal scalp are referred to as the T-complex and include the Ta, a first positive peak at about 100 ms, and the Tb, a subsequent negative going response that peaks at about 160 ms [72, 76]. A fronto-centrally focused negativity that peaks at about 100 ms is referred to as the N1b [43]. Our primary hypothesis was that measures of auditory processing (the N1, Ta, and Tb components of the AEP and lateralization of the Ta and Tb), which provide indices of the integrity of early cortical sensory processing, would be associated with adaptive behavior in the ASD group. While prior studies have focused on a number of different auditory components, we focused on this subset that, based on our and others prior research [6, 7, 48, 75], we expected to be diminished in amplitude in the ASD group compared to the NT group. We additionally hypothesized that the typical rightward lateralization of the auditory response to tones would be reduced in the ASD group.

## Methods

### Participants

The data presented here were collected at the City College of New York and the Albert Einstein College of Medicine over a 10-year period from 2008–2018. Analyses of subsets of the collected dataset have yielded several publications to date [5, 6, 9, 10]. The sample consisted of children and adolescents with ASD (all were verbal) aged 6–17 and a neurotypically (NT) developing sample matched on age and performance IQ (PIQ). This yielded a sample of 107 participants with ASD and 139 NT participants. After participants were excluded due to noisy EEG data, poor performance, or too few trials (detailed in Auditory Event-Related Potential Analysis below), the final sample was 84 ASD participants (72 males, 12 females) and 132 NT participants (62 males, 70 females). Participants were recruited through the Human Clinical Phenotyping Core of the Rose F. Kennedy Intellectual and Developmental Disabilities Research Center, clinician referrals, advertising, and community health fairs. Exclusion criteria included a Performance IQ (PIQ) < 75, abnormal hearing or uncorrected vision, and presence of a neurological disorder. Participants in the NT group were also excluded if they had a neurodevelopmental or neuropsychiatric disorder (as assessed by extensive screening) or had a biological first degree relative with a developmental disorder. Inclusion in the clinical group required an ASD diagnosis confirmed by a trained psychologist, using the Autism Diagnostic Observation Schedule, Second Edition (ADOS-2) [40], the Autism Diagnostic Interview-Revised (ADI-R) parent interview, and clinical judgment. In studies that were conducted before 2012, the first edition of the ADOS was used. Intellectual functioning was measured by the Weschler Abbreviated Scale of Intelligence, Second Edition (WASI-II) [73]. The WASI-II was not administered to 3 ASD and 1 NT participant included in the study. Participants were screened for normal hearing using audiometric threshold evaluation (below 25 dB HL for 500, 1000, 2000, 4000 Hz) performed on both ears using a Beltone Audiometer (Model 112). Vision was assessed first through a phone-screen with the participants’ guardian, and then on-site through Snellen charts. Parents were instructed to refrain from giving their children (*n* = 7 ASD participants) stimulant medication in the 24 h period before the testing session. No participants were taking any other psychoactive medications. Before beginning the study, parents/ legal guardians gave informed written consent, and participants gave verbal or written assent. The Institutional Review Boards of the Albert Einstein College of Medicine, the City College of New York, and the Graduate Center of the City University of New York approved all procedures and were in accord with the ethical standards as stated in the Declaration of Helsinki.

### Clinical measures

Adaptive behavior was measured by the Vineland Adaptive Behavior Scale, Second-Edition parent-report questionnaire, which is an assessment tool that measures adaptive behavior for all ages in the domains of socialization, daily living, communication, motor skills, and maladaptive behavior and is an accepted measure of reported adaptive behavior in ASD [8, 52, 58, 69]. Furthermore, the Vineland is applicable to neurotypically developing children, thus allowing us to determine if adaptive behaviors are correlated with measures of auditory neural processing in both groups. In this study, the socialization, daily living, and communication domains were used for analysis. Motor skills and maladaptive domains were excluded because they were not age-appropriate for all participants (motor skills) and/ or are optional (maladaptive) and were not collected for most participants. We also reported the adaptive behavior composite (ABC) scores from the Vineland, which is a combined score of the socialization, daily living, and communication domains, but did not include this total score in analyses as we wished to examine the specific domains of adaptive behavior.

### Data collection

Clinical and EEG data were collected over 2 visits. On average, the two visits were one month apart (between 1 day and 6-months). In general, clinical data, including the WASI-II and ADOS, were collected during visit 1 to confirm an ASD diagnosis and study eligibility and introduce them to the EEG setup, and EEG recordings were conducted during the second visit. During this visit, the participants performed an audiovisual simple reaction time task while continuous EEG was recorded from 70 scalp electrodes using the BioSemi ActiveTwo™ electrode system, which digitized the data at a rate of 512 Hz and applied anti-aliasing filtering using a first order analog filter (-3 dB at 104 Hz). There were three stimulus conditions presented in random order with equal probability (auditory alone, visual alone, and audiovisual). The “auditory alone” condition consisted of a 1000-Hz tone 75 dBSPL, 5 ms rise/fall time emitted from one speaker (Hartman Multimedia JBL Duet speaker) for 60 ms. The visual only condition was an image of a red circle (3.2 cm diameter) which was displayed on a black background 0.4 cm above central fixation along the vertical meridian on a computer monitor (Dell Ultrasharp 1704FTP) for 60 ms at a viewing distance of 122 cm. The audiovisual condition was comprised of the auditory and visual stimuli at the same time. Stimuli were presented in blocks of 100 trials each, and participants were instructed to press a button on a response pad when they saw the instructed stimuli (circle, tone, or both circle and tone) [5]. The three stimuli were presented randomly with an inter-stimulus interval that varied randomly between 1000–3000 ms. Participants were encouraged to take breaks between blocks to preserve focus and prevent fatigue or restlessness. Participants completed between 9 and 11 blocks (the majority completed 10) of 100 trials each with auditory, visual, and audio-visual stimuli randomly presented in each block. Only auditory-alone trials are considered for the current analyses.

### Auditory event-related potential analysis

EEG data were analyzed using MATLAB (MATLAB r2020b, MathWorks, Natick, MA) and custom in-house scripts. Using Butterworth filter design, a low-pass filter of 45 Hz with a slope of 24 dB/octave, and a high-pass filter of 0.1 Hz with a slope of 12 dB/octave were applied to each participant’s continuous EEG. Bad channels were detected automatically based on joint-probability and kurtosis. A channel was defined as “bad” when recorded data from that electrode exceeded 5 SD from all other electrodes. For eye artifact removal, Independent Component Analysis (ICA) was applied to continuous data high pass filtered at 1 Hz to identify components corresponding to eye movements and blinks. Based on output from an ICA automated component classifier (ICLabel) [54], components with > 80% probability of originating from eye movements and a < 5% probability of origination from a brain source were rejected.

Next, data were re-referenced offline to the common average. Event related potentials (ERPs) for the auditory-alone condition were constructed by dividing the EEG into epochs from -100 to + 600 ms surrounding the onset of an auditory tone. A baseline was defined beginning 50 ms before stimulus onset and ending 10 ms after stimulus onset. An automatic artifact rejection criterion of ± 150 microvolts was used to remove trials containing excessive muscular activity and eyeblinks. An additional artifact rejection threshold was calculated based on an array of maximum amplitudes for each trial (the largest absolute value recorded in a given epoch across all channels); epochs containing values > 3 SD from the median of this array of maximum values were removed. Trials in which participants responded earlier than 100 ms after the auditory tone and trials in which participants responded greater than 1100 ms following the cue were excluded. Participants with fewer than 100 trials remaining after all rejection procedures (TD: 7, ASD: 23) were excluded from analysis. This rejection procedure excluded 12% of our original total dataset (21.5% of ASD group, 5.0% of NT group), leading to our final sample of 84 (72 males, 12 females) ASD participants and 132 (62 males, 70 females) NT participants. For this sample, average number of auditory trials per participant were 203 (SD = 55.3) in the ASD group and 234 trials (SD = 54.1) in the NT group.

The resulting AEPs were referenced to an average of all electrodes. For each participant, electrophysiological indices of early auditory processing were guided by predetermined latency windows over predetermined scalp regions informed by the literature on AEPs [32, 33, 68, 71]. Time-windows and electrodes of interest were confirmed (and adjusted if needed) through inspection of the group-averaged ERPs across the dataset. For statistical testing of group differences, the mean amplitude across the specified time period was calculated for each participant for each component and set of relevant electrodes (see Table 1). For analyses investigating AEP associations with adaptive behavior, the frontocentral N1b was represented by averaging data from the two frontocentrally placed electrodes that best represented the focus of the negative-going N1b response at 100 ms. Lateralization of the AEP was represented by the interaction between left and right temporal scalp regions (left Ta * right Ta; left Tb * right Tb.

**Table 1:** Latency windows and electrodes used for analyses

Component	Latency Window (ms)	Electrodes
Left Ta	100–125	Tp7
Right Ta	100–125	Tp8
N1b	100–125	F1,F2
Left Tb	150–175	Tp7
Right Tb	150–175	Tp8

### Statistical analyses

#### Group differences in auditory responses

Statistical analyses were implemented in R [56]. For each of the three responses of interest (Ta, N1b, and Tb), a repeated measures ANCOVA was run with factors of group (ASD and NT) and, for Ta and Tb, hemisphere. Lateralization was defined as the effect of hemisphere on amplitude. Due to previous studies demonstrating changes in AEP amplitude throughout development in response to auditory stimuli (Pang & Taylor, 2000; [53], age was included as a covariate in the ANCOVAs.

#### *Clinical associations*

To investigate the relationship between auditory responses and adaptive behavior (Vineland socialization, communication, and daily living scores), linear regression models were run for each Vineland domain (socialization, daily living, and communication), with diagnostic group (ASD or NT) and age (due to maturational effects on the Vineland; [20] entered in the first step, and AEP components (Ta, N1b, and Tb) in the second step. These separate models allowed us to better understand potential relationships between early cortical brain processes (as represented by AEP components) and specific domains of adaptive behavior.

## Results

### Descriptive statistics

See Table 2 for age, IQ, and ASD severity of subjects. Independent t-tests demonstrated that there were no significant differences in age or PIQ between the ASD and NT groups (*t* [214] = -0.37, *p* = 0.71, *d* = 0.052) and (*t* [198] = -0.36, *p* = 0.71, *d* = 0.055), respectively. Due to literature suggesting a greater percentage of non-right-handedness in the ASD population [63], and a potential effect of handedness on hemispheric lateralization in response to simple stimuli tasks [49, 66], we examined the frequency of left-handed participants in each group. There was a greater percentage of left-handed participants in the ASD group (14.3%) than in the NT group (6.8%). However, chi-square analysis showed that this difference was not statistically significant, *X*^*2*^ (2, *N* = 196) = 2.63, *p* = 0.27.

**Table 2:** Participant characteristics (mean, range, and standard deviations) after rejection analysis. ASD and NT groups were matched on Performance IQ (PIQ). Verbal IQ (VIQ) and Full-Scale IQ (FSIQ) were significantly different between ASD and NT groups. Severity scores refer to ADOS-2 overall Calibrated Severity Score

	ASD		NT	
	Mean (SD)	Range	Mean (SD)	Range
Total	84 (12 females)	-	132 (70 females)	-
Age	11.4 (2.95)	6.1–17.5	11.5 (3.07)	6.0–17.5
FSIQ	101.5 (17.9)	68–158	109.5 (10.7)	86–142
PIQ	105.4 (17.1)	75–150	104.6 (10.7)	77–134
VIQ	98.0 (20.2)	55–150	111.48 (11.5)	89–141
Severity	7.7 (1.54)	4–10	-	-

While there was a greater percentage of males (85.7%) in the ASD group compared to the NT group (47%0.0), the male to female ratio in this ASD sample is consistent with asymmetry of males to females diagnosed with ASD in the general population [39]. However, this led to an imbalanced sex ratio between the groups. Therefore, clinical and electrophysiological dependent measures were run as a function of sex through independent t-tests and chi-square analyses in both the NT and ASD groups in order to assess whether there was evidence for an influence of sex on the auditory response in the both groups. Sex differences did not attain significance (*p* > 0.05) for any of the dependent measures in the ASD or NT group.

To more fully represent the composition of our study participants, we present additional demographics that are not considered in our analyses. The sample was ethnically and racially diverse, although the largest proportion of participants were White (ASD: 54.8% White, 17.9% Black, 6.0% Asian/Pacific Islander, 1.2% American Indian/Native Hawaiian, 7.1% multiple races, and 13.1% unspecified; NT: 55.3% White, 15.9% Black, 0.8% Asian/Pacific Islander, 10.6% Multiple Races, 17.4% unspecified). Regarding maternal education in the ASD group, 26.2% of mothers had a graduate degree, 33.4% had a college degree, 2.4% had an associate’s degree, 21.3% had a high school diploma or GED, 3.6% had no degree, and 11.9% chose not to answer. For the NT group, 25.0% of mothers had a graduate degree, 23.6% had a college degree, 19.7% had a high school diploma or GED, 9.9% had no degree, and 22.0% elected not to answer. Chi-square analyses did not reveal significant group differences in maternal and paternal level of education or race/ethnicity between groups.

### Behavioral results

Mean hit rates (HR) and reaction times (RT) were averaged across trials for each participant.. The ASD group exhibited longer RTs (ASD = 442.71 ms, NT = 419.06 ms), although this difference was not statistically significant (*t* (214) =  − 1.51, *p* = 0.13,* d* = 0.21). While both groups responded at relatively high rates, the ASD group had lower hit rates (ASD = 89.74%, NT = 92.85%), and this group difference was statistically significant (*t* (214) = 3.59, *p* < 0.001, *d* =  − 0.50).

### Electrophysiological results

**Fig. 1:**
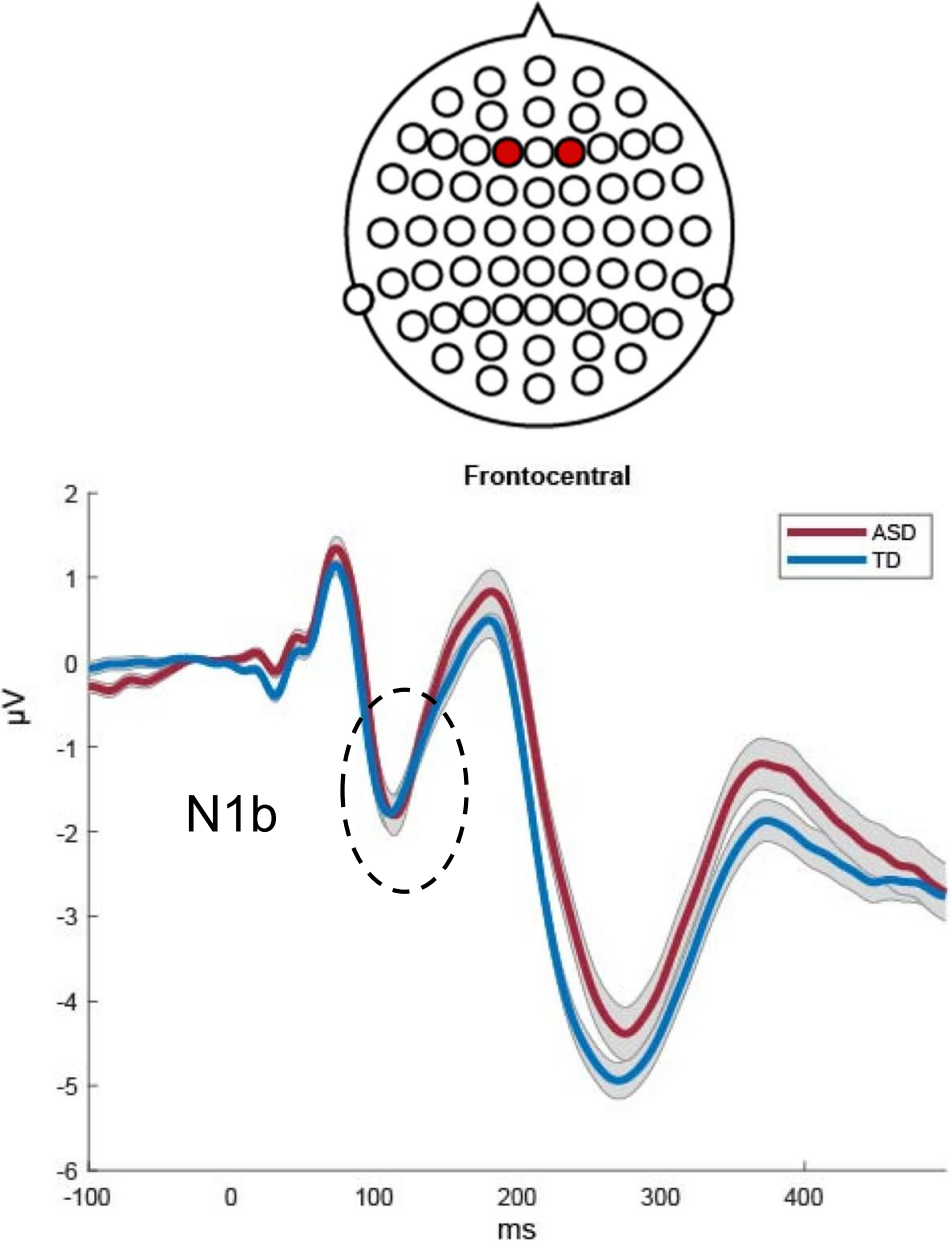
AEP waveforms over frontocentral scalp for each of the groups (average of F1 and F2; as depicted on cartoon of electrode locations)

**Fig. 2:**
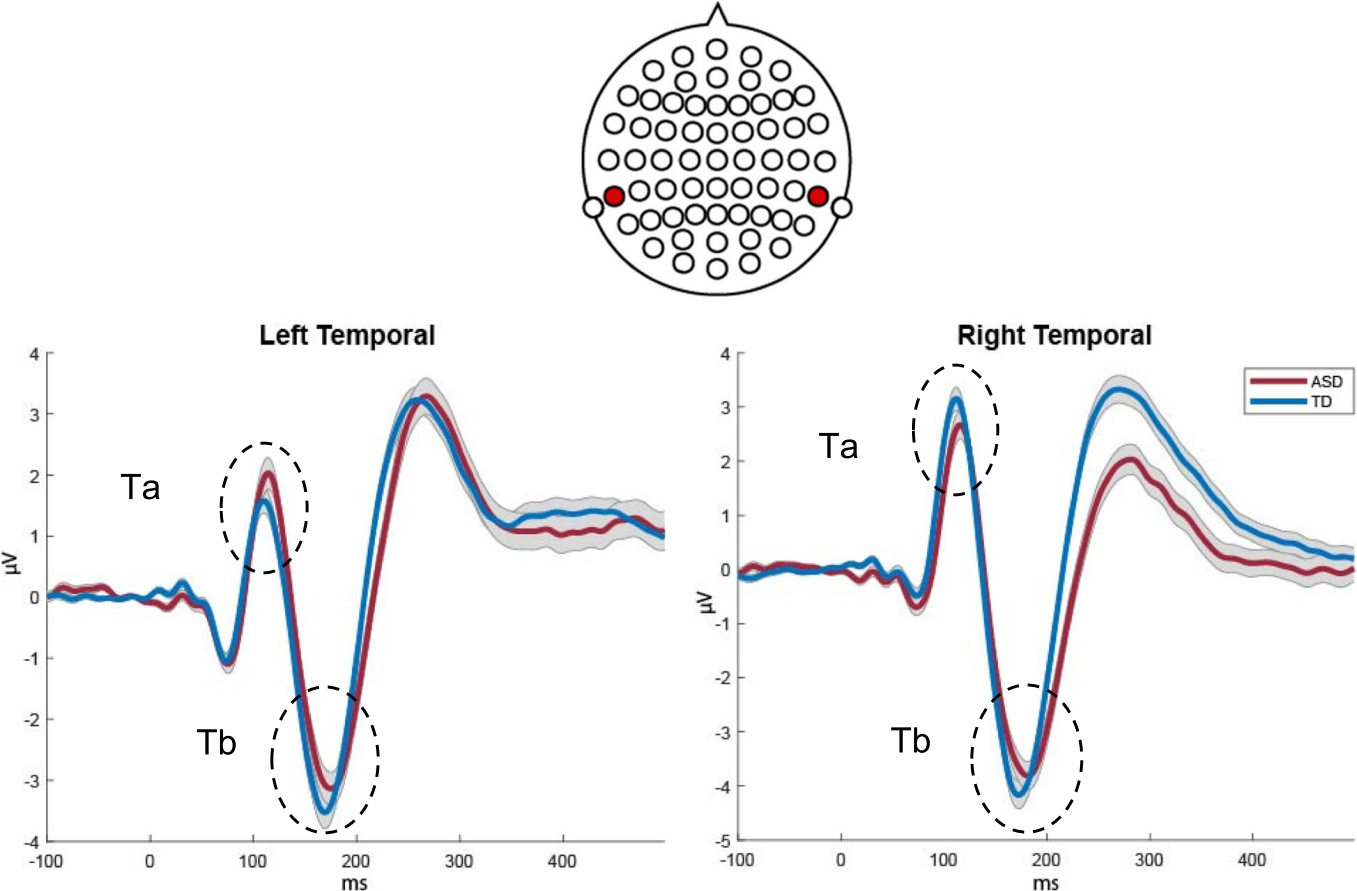
AEP waveforms for both groups over left temporal (Tp7) and right temporal (Tp8) scalp regions

**Fig. 3:**
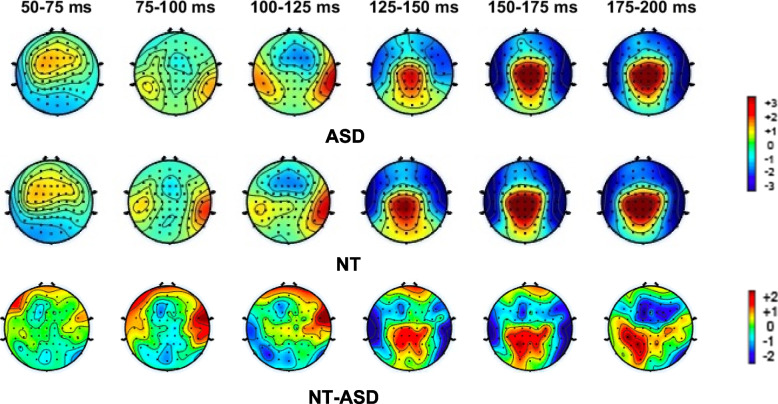
Topography maps depicting average amplitude of the auditory responses in 25 ms steps from 50–200 ms for ASD, NT, and the difference between ASD and NT. The color bar depicts amplitude in μV

The morphology of the AEPs appeared highly similar between the ASD and NT groups (see Figs. 1, 2, and 3), with a positive-going response peaking at ~ 100 ms (Ta) over bilateral temporal scalp regions, a frontocentral negative-going response peaking at ~ 100 ms (N1b), and a bilateral temporal negative-going response peaking at ~ 150 ms (Tb). Nevertheless, visual comparison suggested small group differences in the amplitude of the response over temporal scalp regions. Topographical mapping also suggested rightward lateralization of the response between 75–125 ms in both groups, that appeared to be stronger in the NT group.

#### Group differences

### 100–125 ms window

**Fig. 4:**
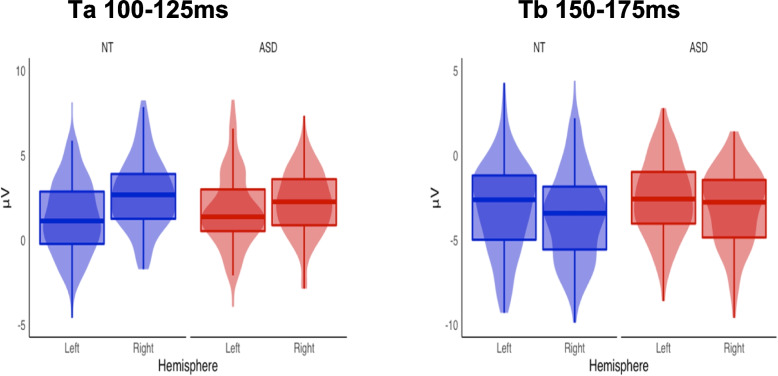
Mean amplitudes of Ta and Tb for ASD and NT groups for left and right hemispheres

All assumptions for the ANCOVA were met. After correcting for multiple comparisons using the Holm-Bonferroni method, the ANCOVA for Ta amplitude revealed a significant main effect of hemisphere, (*F*(1, 424) = 26.81, *p* < 0.001, *η*^2^ = 0.058), reflecting a stronger response over the right compared to left temporal scalp for the total sample. While this was numerically larger in the NT group (see Table 3 and Fig. 4), the group by hemisphere interaction only approached significance (*F*(1, 424) = 18.89, *p* = 0.052, *η*^2^ = 0.0082).For the ANCOVA on the N1b, age was a significant predictor of amplitude (corrected for multiple comparisons), (*F*(1, 212) = 25.80, *p* < 0.001, *η*^2^ = 0.011). This did not interact with group, and group was not significant when age was removed as a covariate.

**Table 3:** Mean amplitudes, standard deviations (SD), and standardized measurement error (SME) of auditory responses over left and right frontocentral and lateral temporal scalp regions (Ta, N1b, and Tb). Additionally, per reviewer request, data for P1 (electrodes FC1, FC2, and FCz) and P2 (electrode Cz) are also reported

Window	Region	Hemisphere	ASD Mean (SD)ASD Mean (SD)	ASD SME	NT Mean (SD)	NT SME
60–100 ms	Frontocentral (P1)	NA	.58 (1.25)	.14	.54 (1.76)	.12
150 -200 ms	Central (P2)	NA	3.65 (2.16)	.24	4.72 (2.38)	.21
100–125 ms	Frontocentral (N1b)	NA	− 1.46 (1.93)	.21	− 1.47(2.02)	.18
	Temporal (Ta)	Left	1.72 (2.27)	.25	1.32 (2.22)	.19
		Right	2.30 (2.21)	.24	2.77 (2.27)	.20
150–175 ms	Temporal (Tb)	Left	− 2.68(2.43)	.27	− 3.21 (2.91)	.25
		Right	− 3.05 (2.28)	.25	− 3.61 (2.75)	.24

### 150–175 ms window

The ANCOVA (adjusted for heteroskedasticity by weighted least squares) over temporal scalp regions in the 150–175 ms window (Tb) revealed a main effect of group (*F*(1, 424) = 6.28, *p* = 0.013, *η*^2^ = 0.010) due to a smaller auditory response over temporal scalp regions in the ASD group. Age was a significant predictor of Tb amplitude (*F(*1, 424) = 8.54, *p* = 0.004, *η*^2^ = 0.015). The group x hemisphere interaction did not reach significance, (*F*(1, 424) = 0.19, *p* = 0.67, *η*^2^ < 0.001).

Table 3 displays mean amplitudes, standard deviations (SD), and standardized measurement error (SME) by group across hemisphere and region for each time frame analyzed.

### Associations between Clinical Measures and Auditory Responses

**Fig. 5:**
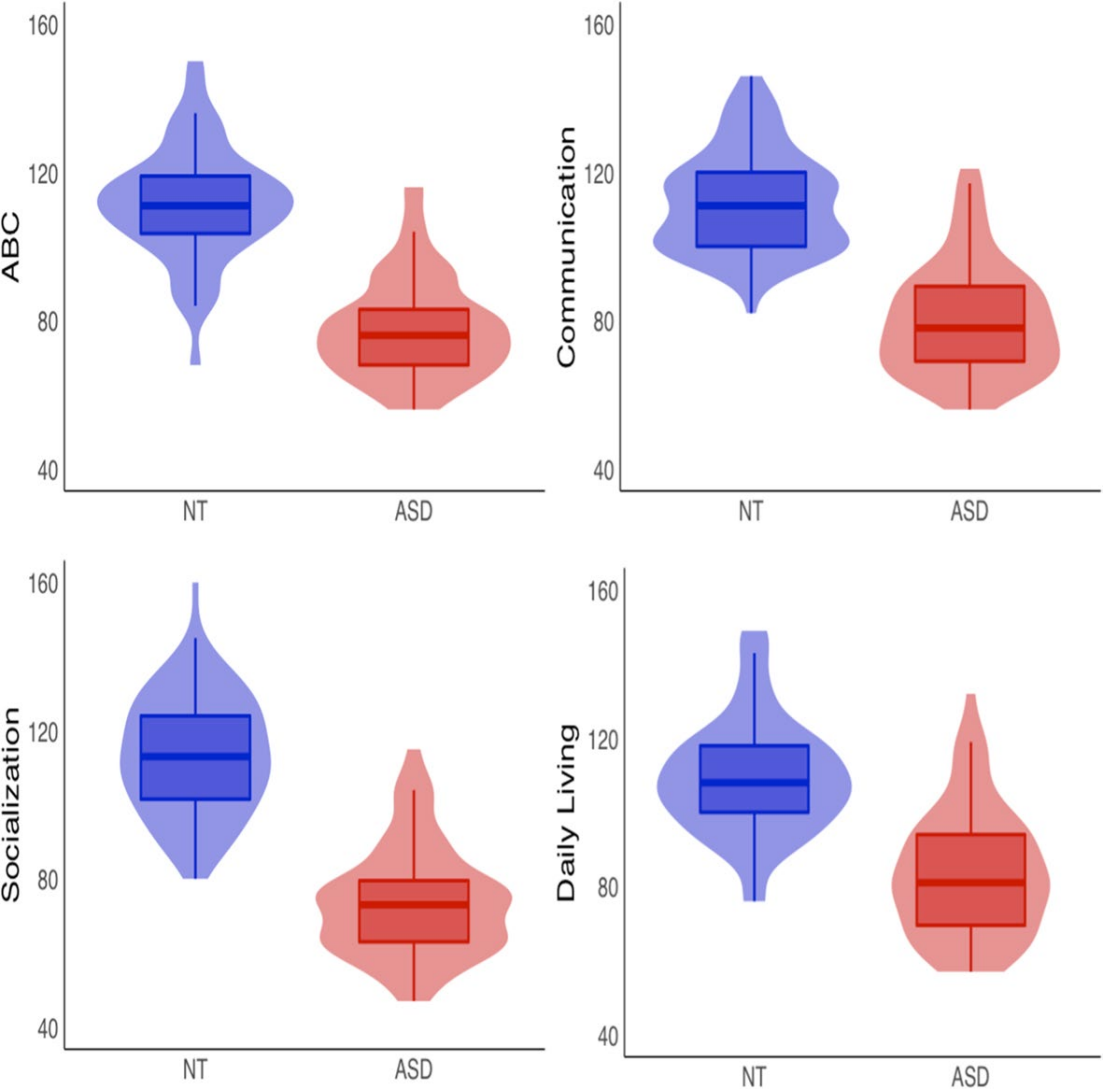
Violin plots of ASD and NT groups for each Vineland domain and total adaptive behavior composite (ABC) score

Twelve participants in the ASD group and 37 participants in the NT group did not have Vineland scores. Therefore, a subset of this sample consisting of 72 ASD and 95 NT participants, matched on age (range of 6.0–17.5) and PIQ (ASD = 105.7; NT = 105.3) was used to investigate the predictive value of measures of auditory responses on adaptive behavior. Chi-square analyses and independent t-tests run between this subset and the participants without Vineland scores did not reveal significant differences on clinical or electrophysiological measures. Within the subset, Vineland scores were significantly lower for the ASD group compared to the NT group for the communication, socialization, and daily living sub-domains, as well as for the total adaptive behavior composite score, all with *p* values < 0.001 (see Fig. 5).

Hierarchical linear regression models with age and group in the first step and AEP components in the second step were run for each Vineland domain across both NT and ASD groups. Separate models were run for each Vineland domain for Ta (left Ta, right Ta, and Ta lateralization, defined as the interaction between left and right Ta), N1b, and Tb (left Tb, right Tb, and Tb lateralization, defined as the interaction between left and right Tb). These models were also run without age to further understand the relationship between the AEP components and Vineland domains when age was not controlled for, and the results remained the same.

#### Vineland socialization

To analyze the relationship between Vineland socialization and Ta components, a linear regression was run with group and age in the first step, and left Ta, right Ta, and Ta lateralization entered in the second step. The model was significant at the first step (*F(*2, 151) = 142.78, *p* < 0.001, f^2^ = 0.65) with age (*B* = -0.16*, t*(150) = -3.42*, p* < 0.001) and group (*B* = -0.79*, t*(150) = -16.53*, p* < 0.001) as significant predictors of Vineland Socialization, explaining 65.4% of the variance. The model remained significant after the inclusion of the Ta components (*F (*5, 148) = 56.36, *p* < 0.001, f^2^ = 0.002), although none of the components were significant at the coefficient level, explaining a non-significant additional 0.20% variance (*p* = 0.88) in Vineland Socialization scores.

When the N1b component was entered in the second step, the model was significant (*F(*3, 150) = 94.88, *p* < 0.001, f^2^ = 0.001), with age (*B* = -0.15*, t*(149) = -3.06*, p* = 0.003) and group (*B* = -0.79*, t*(149) = -16.53*, p* < 0.001) as significant predictors, and N1b contributing only a non-significant additional 0.1% additional variance (*p* = 0.56; 65.5% total variance).

When the Tb components (left Tb, right Tb, and Tb lateralization) were entered in the second step, the model was significant (*F(*5, 148) = 58.87, *p* < 0.001, f^2^ = 0.011), with age (*B* = -0.17*, t*(147) = -3.53*, p* < 0.001), group (*B* = -0.80*, t*(147) = -16.46*, p* < 0.001) and Tb lateralization (*B* = -0.23*, t*(481) = -2.22*, p* = 0.028) as significant predictors of Vineland Socialization, explaining 66.6% of the variance.

#### Vineland daily living

To analyze the association between Vineland Daily Living and Ta components, a linear regression was run with group and age in the first step, and left Ta, right Ta, and Ta lateralization entered in the second step. The model was significant at the first step (*F(*2, 121) = 43.61, *p* < 0.001, f^2^ = 0.42) with age (*B* = -0.18*, t*(120) = -2.65*, p* = 0.009) and group (*B* = -0.61*, t*(120) = -8.85*, p* < 0.001) as significant predictors of Vineland Daily Living, explaining 41.9% of the variance. The model remained significant after the inclusion of the Ta components (*F (*5, 118) = 17.36, *p* < 0.001, f^2^ = 0.005), although none of the components were significant at the coefficient level, explaining a non-significant additional 1.50% variance (*p* = 0.80) in the Vineland Daily Living scores.

When the N1b component was entered in the second step, the model was significant (*F(*3, 120) = 28.83, *p* < 0.001, f^2^ < 0.001), with age (*B* = -0.15*, t*(119) = -3.06*, p* = 0.003) and group (*B* = -0.79*, t*(119) = -16.53*, p* < 0.001) as significant predictors, and N1b contributing 0.0% additional variance (*p* = 0.99; 41.9% total variance).

When the Tb components (left Tb, right Tb, and Tb lateralization) were entered in the second step, the model was significant (*F(*5, 117) = 17.67, *p* < 0.001, f^2^ = 0.009), with age (*B* = -0.19*, t*(117) = -2.70*, p* = 0.008) and group (*B* = -0.80*, t*(117) = -8.74*, p* < 0.001) as significant predictors of Vineland Daily Living. The Tb components contributed a nonsignificant additional 0.9% of variance (*p* = 0.60) in Vineland Daily Living scores.

#### Vineland communication

For the regression models between Vineland Communication and Ta components, the model was significant at the first step (*F(*2, 164) = 111.68, *p* < 0.001, f^2^ = 0.58) with age (*B* = -0.13*, t*(163) = -2.55*, p* = 0.012) and group (*B* = -0.75*, t*(163) = -14.68*, p* < 0.001) as significant predictors of Vineland Daily Living, explaining 57.7% of the variance. The model remained significant after the inclusion of the Ta components (*F (*5, 161) = 44.03, *p* < 0.001, f^2^ = 0.001), although none of the components were significant at the coefficient level, explaining a non-significant additional 0.10% variance (*p* = 0.94) in Vineland Communication scores.

When the N1b component was entered in the second step, the model was significant (*F(*3, 163) = 74.00, *p* < 0.001, f^2^ < 0.001), with age (*B* = -0.13*, t*(162) = -2.55*, p* = 0.012) and group (*B* = -0.75*, t*(162) = -14.68*, p* < 0.001) as significant predictors, and N1b contributing 0.0% additional variance (*p* = 0.99; 57.7% total variance).

When the Tb components (left Tb, right Tb, and Tb lateralization) were entered in the second step, the model was significant (*F(*5, 161) = 45.03, *p* < 0.001, f^2^ = 0.006), with age (*B* = -0.13*, t*(160) = -2.55*, p* = 0.012) and group (*B* = -0.75*, t*(160) = -14.68*, p* < 0.001) as significant predictors of Vineland Daily Living. The Tb components contributed a nonsignificant additional 0.6% of variance (*p* = 0.48) in Vineland Daily Living scores.

**Fig. 6:**
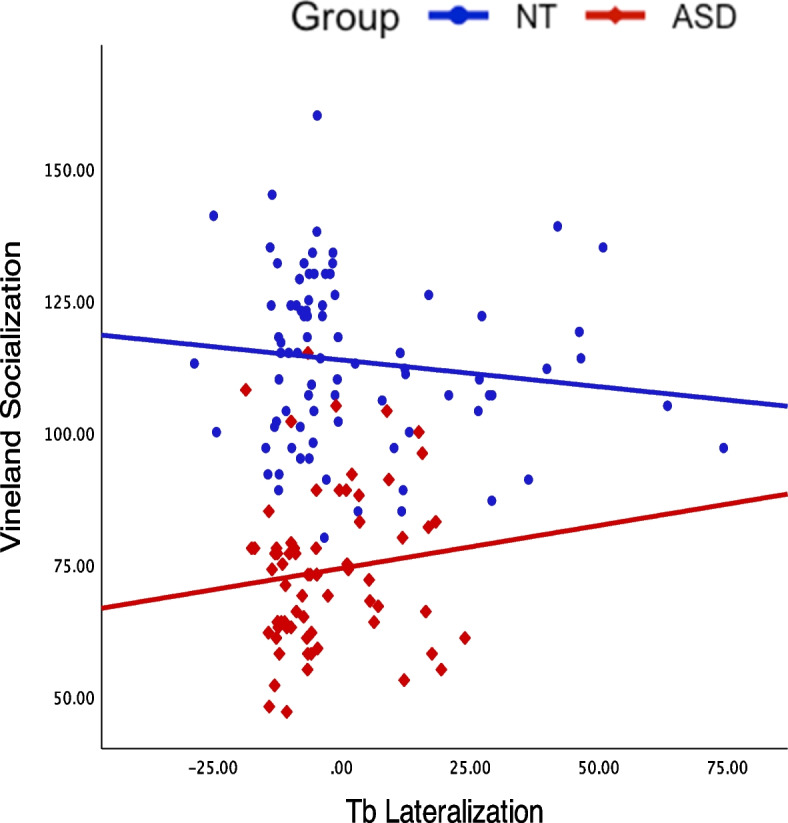
Scatter plots of Tb lateralization and Vineland Socialization

See Fig. 6 for scatter plots between Ta lateralization responses and Vineland domains by group.

## Discussion

Engagement in age appropriate adaptive behaviors in everyday situations is significantly reduced in ASD [1]. Prior research on electrophysiological responses to auditory stimuli suggests that children and adolescents with ASD exhibit atypical auditory responses [6, 7, 27, 48, 70]. It is reasonable to assume that altered brain responses in a clinical group are related to aspects of the behavioral phenotype. For example, altered auditory processing has been linked to autism severity [5]. However, how auditory cortical responses are related to adaptive behaviors in autism has not been extensively studied. Here, in an analysis of this relationship in a relatively large sample of participants, we found that greater rightward lateralization of the early AEP to tones was associated with better Vineland socialization adaptive scores in ASD.

With regard to group differences, the ASD group exhibited significantly diminished AEP responses to tones in the 150–175 ms timeframe over bilateral temporal scalp regions, corresponding to the Tb component. Diminished AEPs align with our previous work [6] as well as findings from other groups in which responses between 100 and 200 ms were of smaller amplitude in ASD compared to control groups [7, 48, 75]. In the earlier epoch (100–125 ms) of analysis, for the Ta, rightward lateralization of the response over temporal scalp regions was observed in both the ASD and the TD groups, whereas a group by lateralization interaction failed to reach significance, and no group differences were observed for the fronto-centrally focused N1. The lack of a significant group effect over temporal regions in the early time frame conflicts with some other studies (see meta-analysis of [75], and may be explained in part by the large age-range considered in the current analysis, which would be expected to increase intersubject variability of the AEP and lead to greater variance in the dependent measures. Consistent with previous studies [6, 22, 53], age was significantly related to the N1b response as well as the Tb response; however neither of these responses interacted with group, thus failing to reveal an interaction between childhood development and atypical auditory processing in ASD.

Both groups exhibited the expected rightward lateralization of the AEP to tones. While this lateralization appeared to be reduced in the ASD group (see the 100–125 ms and 150–175 ms topography maps in Fig. 3), this difference did not hold up to statistical testing in our primary analyses. Tellingly, however, the lateralization index derived from the same data revealed a significant relationship (although notably a relatively small effect size) with adaptive behavior in the ASD group (See Fig. 6). This suggests that lateralization is an informative biomarker of altered functioning level in ASD. Altered lateralization in ASD has been previously observed in visual object processing, language, and motor studies (see e.g., [16, 17, 34, 36, 45, 51]. Roberts and colleagues identified hemisphere specific processing differences for tones in ASD, finding a delay in the magnetic auditory evoked response to tones that was more robust in right compared to left auditory cortex [60]. The finding of altered lateralization across sensory and motor processing and for both language and non-language stimuli suggests that the hemispheric specialization that is seen in typical development [18] is reduced in ASD. As such, differences in lateralization may be a common feature of altered neurodevelopment in ASD. Importantly, in line with the idea that greater extent of atypical neural development is likely to be associated with greater clinical severity, our analyses revealed that greater lateralization of responses to auditory tones was associated with better adaptive functioning in the socialization domain in individuals with ASD.

Why might disruption of auditory processing lead to behavioral and perceptual/cognitive sequelae, and in particular delayed development of adaptive behavior? On the one hand, altered auditory responses could represent early indices of disrupted cortical pathways and processing, regardless of stimulation type. On the other hand, the Vineland socialization domain includes items such as engaging in play, conversation, friendships and coping skills; altered auditory brain responses and specific auditory sensitivities in a child with ASD may make these activities more difficult, preventing them from efficiently developing appropriate adaptive skills. Indeed, hyper- or hypo-reactivity to sensory stimuli is characteristic of ASD [1], and is associated with poorer adaptive behavior [15, 31, 46, 61]. At the same time, it is important to mention that the Vineland scales do not directly measure auditory-specific adaptive functioning, and thus the associations between the observed AEPs and adaptive behavior scores could also be driven by another unknown variable such as sensory hyper-/hypo-sensitivity (but see [5]. Clearly more research is needed to unpack the respective roles of altered neurodevelopment and atypical auditory processing for adaptive skills. Furthermore, in this study, only specific AEPs were examined; future research should examine potential relationships between adaptive behavior and other features of the auditory response, such as the P100, P2, or, given the appropriate paradigm, the mismatch negativity (MMN). It is also important to note that while we found a significant association between later temporal responses and Vineland socialization, these effects were small. Given this small effect size, more research is warranted before drawing strong conclusions on the relationship between auditory brain responses and adaptive behavior.

Although a large number of studies investigating early auditory cortical processing in ASD have demonstrated atypical responses in ASD groups compared to NT groups [6, 7, 27, 41, 42, 48, 60, 70], these group differences are often modest and the specific results are heterogeneous across studies [75], with some studies finding little or no differences [29]. ASD has a strong genetic basis that is, in most cases, polygenic and variable across individuals [57], with risk associated allelic variants that implicate neurobiological pathways involved in fetal neural development and synaptic function [12]. Consistent with this heterogeneity, prior work suggests that atypical connectivity in sensory and higher order networks in ASD is highly idiosyncratic compared to controls [2, 25]. Thus, while the auditory cortex appears particularly vulnerable to resulting neuropathology, just how this plays out may vary based on an individual’s genetic background, specific set of genetic vulnerabilities, and the environmental factors that they are exposed to. Variability in how auditory neurophysiology is altered across individuals with autism could interfere with the ability to identify a good singular auditory biomarker for adaptive behavior.

## Conclusions

This study supports prior findings that children and adolescents with ASD exhibit atypical auditory responses at the neural level. Furthermore, our results support a relationship between atypical auditory processing in children and adolescents with ASD and adaptive behavior. Although there is great heterogeneity in the ASD population, these findings utilizing a large dataset with a wide range of ages indicate the presence of a relationship between basic neuropathological processes and maladaptive behavior in this population and reveal the potential of functional lateralization to serve as a biomarker of phenotype in ASD. Future studies will be needed to understand if and how this knowledge can inform approaches to improving adaptive function in this group, especially in the domain of socialization. Furthermore, expanding the group to include minimally verbal and nonverbal individuals with ASD will be an important step in the identification of biomarkers of adaptive behavior and other aspects of the autism clinical phenotype.

## Data Availability

Data from the findings of this study are available from the authors upon request. The authors will make the Matlab scripts available in a public repository (Github).

## Abbreviations

ADI-R: Autism Diagnostic Interview-Revised
AEMF: Auditory evoked magnetic fields
ASD: Autism spectrum disorder
ADOS-2: Autism Diagnostic Observation Schedule-2
EEG: Electroencephalography
ERP: Event related potential
FSIQ: Full-scale intelligence quotient
MEG: Magnetoencephalography
NT: Neurotypical
PIQ: Performance intelligence quotient
VIQ: Verbal intelligence quotient
WASI-II: Weschler Abbreviated Scale of Intelligence, Second Edition
AEP: Auditory Evoked Potential
SCP: Statistical Cluster Plot
ABC: Adaptive Behavior Composite
RT: Reaction Time
HR: Hit Rate
